# Work, family or personal life: Why not all three?

**DOI:** 10.4103/0019-5545.74301

**Published:** 2010

**Authors:** T. S. Sathyanarayana Rao, Vishal Indla

**Affiliations:** Department of Psychiatry, JSS University, JSS Medical College Hospital, Mysore - 570 004, India; 1 Chief Psychiatrist, Vijayawada Institute of Mental Health and Neurosciences, Vijayawada - 520 002, India

Work is taking over the lives of many of us in today’s fast-paced, global environment, and if we do not guard ourselves against work–life imbalance, there could be increasing work–family conflicts and stress resulting from long hours and workload escalation. Vacations are getting shorter and are often clubbed with work, or even worse, many do not have the time for a vacation. Quality family time is getting invaded by the omnipresence of media and the internet. It has been well established that most adults suffer adverse health effects from stress, and 75–90% of all physician office visits are for stress-related ailments and complaints. Stress is linked to the six leading causes of death: heart disease, cancer, lung ailments, accidents, cirrhosis of the liver and suicide.[1] People who experience stress typically go through different stages and degrees of suffering and along the way they pass on their stress to their direct environment, their families, co-workers and friends. Research in the field of work and family has well established the spillover and crossover effects of stress affecting co-workers, spouses, children, and the community at large.[2] Decrease in work–life balance has been linked to higher unwanted turnover, lower physical and psychological well-being, lower productivity, greater stress-related ailments, and the like. The Waste is immeasurable.

## MYTH OF THE IDEAL EMPLOYEE

Myth of an ideal employee perpetuated by the society creates intense time pressure, or what some refer to as a time famine,[3,4] which can lead to stress and job dissatisfaction, possibly creating work–family conflict. The male model of work prescribes an ideal employee who is male, full-time, and continuously at work from the end of the education, fully committed to the organization, and without any responsibilities outside of work.[5] This model is no longer valid and has become outdated.[6] In addition, we can also observe a change in attitudes toward what constitutes a successful career, especially among the newer generations. The current generation started to question old assumptions about how work is done, how to show commitment, where and when to work, and how to advance in the company. Along with having a highly paid job, they strive for a more “complete” life that includes both a successful professional and a personal life. Organizations that monopolize the time of employees challenge the ability of employees to perform well in other important roles within the family and the community.

The cover of Fortune’s November 2005 issue was dedicated to the stress and burnout of the most elite group of employees within organizations today – the senior executive level. In this issue, senior executives were surveyed about their issues of work–life balance. While 49% of respondents were self-confessed workaholics, 64% of respondents stated that at this stage of life, they would choose more time over money. The most profound result was that 87%of the respondents agreed that the companies that restructure senior management jobs in ways that would both increase productivity and make more time for a life outside the office would have a competitive advantage in attracting talent.

## CHANGING DEMOGRAPHICS AND THE IMPACT ON WORK–LIFE BALANCE

The percentage of women in the active work population has increased rapidly in many countries around the world, including ours. As a consequence, we have seen the proliferation of dual-income families where role expectations toward men and women, both in their work activities and their domestic responsibilities, have radically changed.[7] Apart from the many positive effects of women’s integration into the work force, like the increase in nations’ productivity, the wealth and consumption power of families, the financial independence of women, and an improvement of gender equity, there are some negatives in the form of pressure on family time. Due to this new mix of gender equity, shifting role expectations, and family time scarcity, many men and women are required to find new ways to balance their professional and personal lives. Judging from the high rate of separations and divorces, many couples seem to struggle with the new reality. Separations of couples have resulted in an increasing prevalence of new family forms, like mono-parental and mixed families, in which two single parents together raise their children of previous marriages. In these families, working men and women are experiencing increasing levels of work–family conflict.

Couples have started to postpone and control their procreative activity, resulting in an increasing average first childbearing age and a considerable reduction in fertility. These demographic trends suggest that individuals have less of a traditional support at home, i.e., one spouse taking care of the home, less of a child centered family life (i.e., children being a diversion from work) and more work centrality, especially among well-educated career professionals (i.e., self-worth may be originating more from work roles than other life roles). In other words, changes in workplace demographics may have created the potential for a strain on work–life balance and burn out. Some of these changes in the Indian family structure are amply reflected by the national consensus data. According to the 1981 census, the population growth was higher than the growth of households, a phenomenon which saw a turnaround in 1991 census which showed that the number of households grew at a faster pace than the population and this trend gathered further strength in 2001 census data. This perhaps indicates that nuclearization of families is growing in the society which is more evident in urban areas than in the rural, although happening in both the settings.[8]

## VARIOUS CULTURAL PERSPECTIVES ON WORK–LIFE BALANCE

Cultures differ on the extent to which they focus on career success over quality of life or vice versa. In some cultures like ours, we are more likely to observe people who opt to work on weekends, take fewer and shorter vacations, answer emails and make work-related calls from home, while some of the western societies believe that different spheres of one’s life may co-exist but should not interfere with each other. Also, additional compensation is not as motivating a reward as compared to additional vacation, in many of the developed economies, whereas the contrary is true for most of the developing economies like ours. The country with the lowest average annual hours worked is the Netherlands where the average employee works 1368 hours per year or the equivalent of 35 hours per week for 39 weeks of the year.[9] In India, though the working hours of various professions vary hugely, it can safely be said that the working hours are much more than in the west.

The most important barrier, and probably the most difficult to overcome, is an unsupportive culture created by the underlying assumptions of the primacy of work.[10,11] These underlying assumptions create a work culture that prioritizes work over family, that rewards the “ideal worker” who will work long hours and meet client demands at all costs, and that equates productivity with time expended.[12,13] If we look at the history of how countries and work cultures evolve, it is interesting to note how people and policies initially focus excessively on the primacy of work during the boom phase of economy and then when the economy gets saturated, policies and the cultures shift toward enriching work–life balance. Japan is a prime example of this. In the 1970s and 1980s, Japan was derided for being nothing but a nation of workaholics who needed to get a life. But now, Japanese are a changed lot. More and more salary men are deciding not to go drinking with the boss. Some, heeding government pleas for a greater work–life balance, are focusing on their homes and their hobbies, while others are taking sabbaticals or even dropping out of corporate life. Japanese women have decided not to have babies in a society where children mean the end of a career, the end of independence and a cut-throat struggle to get into the best kindergarten, then the best school and the best university.

The average life expectancy in Japan at 82 years is one of the highest anywhere, while crime levels are among the lowest. The nation’s artistic life – from Kabuki to cinema – is exquisite and world famous. Income disparities are low. Tokyo’s 160,000 restaurants, for instance, boast more Michelin stars between them than those of Paris and New York City combined. So, while legions of salary men across the world are working themselves to death, plenty of Japanese are indulging their appetite for life.[14]

## IMPACT OF TECHNOLOGY ON WORK–LIFE BALANCE

Parallel to these changes in the workforce, work itself has undergone major changes over the last decades. Technology has created a sense that life is moving faster and that more and more activities are squeezed into shorter amounts of time. New technologies have made it possible to perform job tasks from everywhere at any time and have increased the number of interruptions during work as well as expectations of speedy replies, fragmenting time and indirectly, affecting productivity and also diminishing personal space and time. Many of us feel increasingly pressured to not only work faster but also work longer hours. While there is a tendency to think that the new array of gadgets that we are surrounded by in our daily life is a boon, the contrary may be true. The world would be more efficient, more educated, if we control technology and the technology does not control us.

## IMPLICATIONS OF WORK–LIFE BALANCE

Individuals experiencing greater work–life balance have better health and wellness, greater organizational commitment, greater job satisfaction, better goal achievement, and family happiness. At the family level, work-life balance promotes greater marital and family stability, family cohesion, and marital and family happiness. Work–life balance reduces turnover, improves performance, and lowers the incidences of lateness and absenteeism. All of us should strive for policies and practices that create an enriching working environment. In the end, optimizing the harmony between the different spheres of life serves multiple purposes: economic, social, and ethical. Recent initiatives in this direction are on-site day care centers/creches which are convenient for employees with kids. Help from the organization with the time consuming and the less desirable chores like picking up the dry cleaning, going grocery shopping, paying bills can go a long way in improving productivity and Work–life balance.

To reduce the detrimental effects of a sedentary life style, many organizations are now equipping themselves with fitness centers that employees can use on work time to relieve stress, and a staff of doctors, nurses, and physical therapists available to the employees at any time, all at no expense to the employee. As a result of these Work–life balance implementations, the organizations enjoy an extremely low turnover rate of 3%, low absenteeism, and high employee and customer satisfaction.

## CONCLUSIONS

Work, family and personal life should be complimentary to each other and not conflicting with each other. Some are successful in their careers but fail in family and personal life, whereas some others who have a vibrant personal and family life are below par at work. Being successful in one sphere of life at the cost of the other is not a healthy sign. In the long run, family happiness and a decent personal life are key determinants of a successful career. A balancing act among these domains may not be as easy as we think, but a sincere attempt in this direction will definitely yield fruitful results.

As professionals engaged in the mental health of people, it is important to consider Work–life balance as a priority issue and make appropriate changes in the working conditions, thereby not only increasing the long-term productivity of communities but also protecting the social fabric of our society against irreversible damage. As stated by Xerox Corporation CEO Anne Mulacahy, “Businesses need to be 24/7,… individuals don’t.” So, when is your next vacation?
